# Three-Dimensional Displacement Measurement of Micro-Milling Tool Based on Fiber Array Encoding

**DOI:** 10.3390/mi14030631

**Published:** 2023-03-10

**Authors:** Binghui Jia, Min Zhang

**Affiliations:** School of Mechanical Engineering, Nanjing Institute of Technology, Nanjing 211167, China

**Keywords:** micro-milling tool, vibration, optical fiber sensor, encoding

## Abstract

The vibration of the micro-milling tool presents a significant chaotic vibration phenomenon, which has a great influence on the tool life and part machining precision, and is one of the basic problems restricting the improvement of machining efficiency and machining accuracy in micro-milling. To overcome the difficulty of the traditional vibration measurement method with the online measurement of micro-milling tool multi-dimensional vibration, a three-dimensional (3D) measurement method of the micro-milling tool is proposed based on multi-fiber array coding, which converts the tool space motion into a decoding process of the optical coding array employing the tool modulating the multi-fiber array encoding. A 6 × 6 optical fiber array was designed, and a 3D motion platform for micro-milling tools was built to verify the characteristics of the optical fiber measurement system. The measurement results show that the measuring accuracy of the system reached 1 µm, and the maximum linear error in x-, y-, and z-direction are 1.5%, 2.58%, and 2.43%, respectively; the tool space motion position measurement results show that the maximum measurement error of the measuring system was 3.4%. The designed system has unique coding characteristics for the tool position in the space of 100 µm^3^. It provides a new idea and realization means for the online vibration measurement of micro-milling tools.

## 1. Introduction

Micro-milling usually refers to the micromechanical cutting technology that uses ultra-high-speed (UHS) spindles (>60,000 revolutions per minute) with micro-scale tools. Usually, the workpiece feature size is 10 μm–1 mm, and the diameter of the micro-milling tool is less than 1 mm. It has the advantage of machining complex three-dimensional surfaces of steel, titanium alloy, stainless steel, aluminum alloy, ceramics, and other non-metallic materials. Micro-milling overcomes the shortcomings of traditional precision and micro-manufacturing processes. It is not only a key technology to realize the machining of high-performance micro-structure parts, such as aerospace, robots, and medical instruments, but also a research frontier and application hotspot in the field of precision and ultra-precision machining around the world [1,2]. Compared with traditional milling technology, the micro-milling tool not only is reduced in size by a large scale but also removes the material discontinuously on a sub-micron or micron scale under a super-high speed. Hence, the motion of the micro-milling tool is affected by a comprehensive influence of size effect, tool runout, installation error, and spindle speed. It brings some disadvantages as well, such as a more complex material-removal mechanism, difficult modeling process, and obvious chaotic vibration phenomenon in the processing of micro-milling [3]. Because a very small tool is used in the micro-milling process, a low speed for material processing may cause tool damage or fracture more easily, as the tool must provide enough force to complete the removal of materials, and in this case, the force between the workpiece and the tip often exceeds the yield limit of the tool. To this end, high speed is required to increase the gyroscopic moment required to remove material. The micro-milling tool also provides better surface finish, high material removal rate, and reduced cutting forces. However, this introduces new problems [4,5,6]. Among them, the solution of the tool vibration problem is particularly urgent, as it has a great potential to harm the tool life and workpiece machining accuracy, which is one of the basic problems restricting the improvement of machining efficiency and machining accuracy in micro-milling [7,8], The tool displacement variation at ultra-high speed can be more than 15 microns because of radial throw and other comprehensive effects [9]. In general, the establishment of a frequency response model of the tooltip or the online monitoring of tool vibration is considered to be the preliminary foundation for mastering the vibration of micro-milling tools [10]. However, the realization of these two methods requires a precise and reliable tool displacement measurement system [11].

The acquisition of a tooltip frequency response function (FRF) in micro-milling plays an important role in establishing accurate and reliable stability lobule diagrams (SLD). Experimental modal analysis (EMA) is the most used method for tool FRF determination [12]. Experimental modal analysis technology combines theoretical analysis with dynamic testing through system identification theory and modal analysis theory, which generally has three basic links: excitation, measurement, and analysis. The frequency response function of the tool system is determined by measuring the exciting force and tool vibration response at the same time when the exciting force acts on a certain point of the micro-milling system. However, the small size and weak rigidity of micro-milling tools often make it difficult for researchers to obtain tool vibration signals. In order to make the process more reliable and less time-consuming, Joel Martins Crichigno Filho [13] investigated a device that could facilitate the positioning of the laser vibrometer beam directly at certain points on the micro-milling tool; usually, it was not feasible to carry out a hammer experiment directly at the micro-tooltip. Shivang Shekhar [14] proposed a novel modal-Tchebychev domain coupling technique for coupling the spindle and microtool dynamics, and it was used to predict the tooltip dynamics accurately in micromachining when using ultra-high-speed spindles and arbitrary microtool geometries. In practical application, despite the sensors installation or the impact location of the hammer having a great influence on the tool’s dynamic characteristics, the micro-tool was separated into two parts by Peng Wang based on the receptance coupling method. The dynamic parameters of one part (the tool head) were obtained with a modal test, and the dynamic parameters of the other part (the tooltip) were obtained from finite element analysis [15]. These studies undoubtedly play an important role in revealing the vibration characteristics of micro-milling tools and improving the machining stability of micro-milling. However, the size effect, tool runout, installation error, micro friction, and so on are key factors that affect the vibration of the micro-milling tool. The uncertainty caused by the variations in tool structure size and workpiece material brings challenges to the efficient promotion of this method, which obtains tool vibration characteristics through offline vibration measurement in advance [16].

Micro-milling vibration monitoring is a hot technology for enterprises to improve their equipment competitiveness and a frontier and difficult point in modern science and technology academic research [17,18,19]. Laser displacement sensors [9,13,14,20], microphones [21], capacitance sensors [22], acceleration sensors [23], and three-dimensional force sensors [24,25,26,27] are often used by researchers to obtain machining state signals or use multi-sensor information fusion based on the combining of some of these sensors to predict the tool vibration state [9,20,22]. However, there are some problems when the laser displacement sensor is used for the measurements of micro-milling vibration, such as when the spot is larger than the surface area of the tooltip and the sensor needs to be installed far away from the tool system [28]. The sensitivity of the microphone is affected by many factors, such as directivity, bandwidth, and environment, so it is a great challenge when acquiring weak chatter signals in the early stage. Although the force sensor can directly reflect the cutting state of the tool, the cutting force in ultra-precision micro-milling is usually very weak (even to the mN level). In addition, the extremely high spindle speed makes the cutting force in micro-milling close to the natural frequency of the force sensing system, and useful tool vibration information can be seriously interfered with or even submerged. The multi-sensor information fusion method overcomes the limitation of signal acquisition using a single sensor in the micro-milling process, but the system is usually very large and not good for installation in micro-milling equipment, which limits its application in the online measurement of the machining process [17,29].

In contrast, the optical fiber sensing system is small in size, light in weight, high in measurement accuracy, corrosion resistant, high-temperature resistant, strong in anti-electromagnetic interference and atomic radiation performance, and easy to miniaturize the design. It is an ideal object for realizing high-precision parameter sensing [30,31,32,33] and has a unique advantage in the application of parameter measurement in the machining process [34]. Two laser Doppler vibrometers (LDVs) were used by O. Burak Ozdoganlar to measure the radial motion of the attached microtool [9]. XU Jinkai [35] proposed a three-dimensional pose reconstruction method based on the depth of field of the micro-milling tool. A non-contact, single-sensor system and a signal processing algorithm were presented by Robert Kuschmierz for vibrational behavior measurements [8]. However, because a large number of lenses were used, the measurement system was more complicated. A measurement method for the 3D motion of a micro-milling tool with a simple sensing structure is relatively rare. With the development of optical fiber technology, it is possible to encode arbitrary complex image information by constructing an optical fiber array [36], even realizing the active photoelectric conversion and electro-optic modulation [37]. The spatial dimension is introduced based on the regulation of the optical fiber spatial light field, so the information carried by the signal can be superimposed onto the carrier optical field. Part of the information is hidden in the fiber index number, making the fiber index number become an additional data-carrying mode with stronger information transmission ability [38,39].

In this paper, a 3D displacement measurement method of the micro-milling tool is proposed based on multi-fiber array coding. The tool space motion is converted into the decoding process of the optical coding array through the tool modulation of the multi-fiber array encoding, which provides a new idea and realization means for the online displacement measurement of the micro-milling tool. Compared with conventional measurement techniques, the proposed measurement system offers the advantages of (1) in situ, simultaneous 3D measurements for micron displacement with only one sensor head; (2) no special requirement for tool reflection surface size and direct measurements at milling tooltip; and (3) tool motion coded directly using an optical fiber array and reflected light intensity with no interferences of electromagnetic and speckle noise.

## 2. Methodology

**Figure 1:**
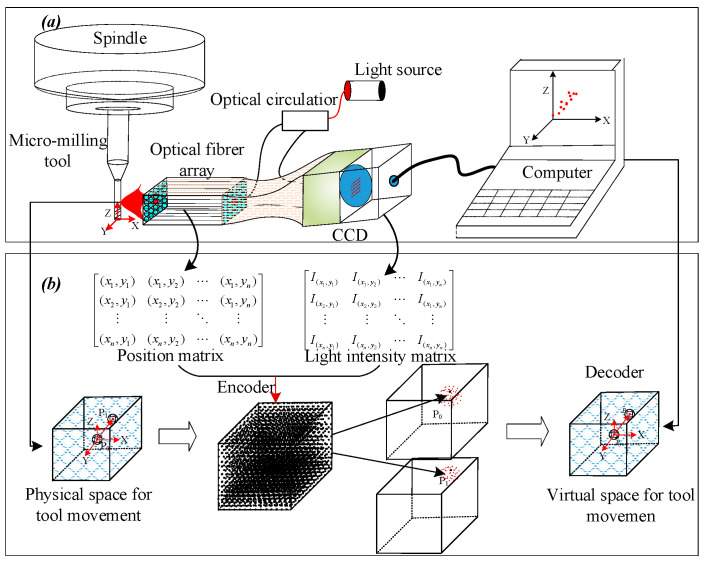
Principle of micro-milling tool vibration measurements. (**a**) Composition of tool vibration measurement system. (**b**) Principle of the measurement system.

The composition of the measurement system is shown in Figure 1a. It mainly consists of an optical fiber array, a light source, a photoelectric conversion device (CCD), and a computer. Figure 1b shows the basic principle of optical fiber measurement. The motion of the micro-milling tool is essentially the change of the tool to different positions in a certain space. Therefore, it is assumed that the tool has a unique coding mark at each position. When the tool moves to a certain position, the coding of the position can be obtained in time, and the measurement of the tool motion can be realized by analyzing the coding changes. Based on this idea, a tool vibration measurement method based on fiber array coding is proposed in this paper. As shown in Figure 1a, one fiber of the fiber array is used as the transmitting fiber, and other fibers are used as the receiving fiber. The light is irradiated on the tool surface through the transmitting fiber emitted from the light source, and the reflected light is received by the receiving fiber. When the tool is moving, the light spot reflected by the tool surface changes with the change of the tool position, and the receiving intensity of each sub-fiber in the fiber array changes with the tool movement. As the spot position and light intensity on the receiving end face of the fiber array change synchronously with the tool movement, the position of each sub-fiber and the reflected light intensity of each point in the fiber array can be used as coding to identify the possible moving space of the tool. As shown in Figure 1b, the relative position of each sub-fiber is represented by the position matrix (based on the design and known quantity), and the light intensity received by each sub-fiber is represented by the light intensity matrix (measured by photoelectric conversion devices). The combination of the position matrix and the light intensity matrix forms the spatial coding of the tool’s possible motion region. When the tool moves, the spatial encoding constructed by the position matrix and the light intensity matrix changes accordingly. The tool vibration measurement is realized by decoding the encoding space. In this method, the tool vibration is transformed into spatial coding matrix analysis using an optical fiber array.

**Figure 2:**
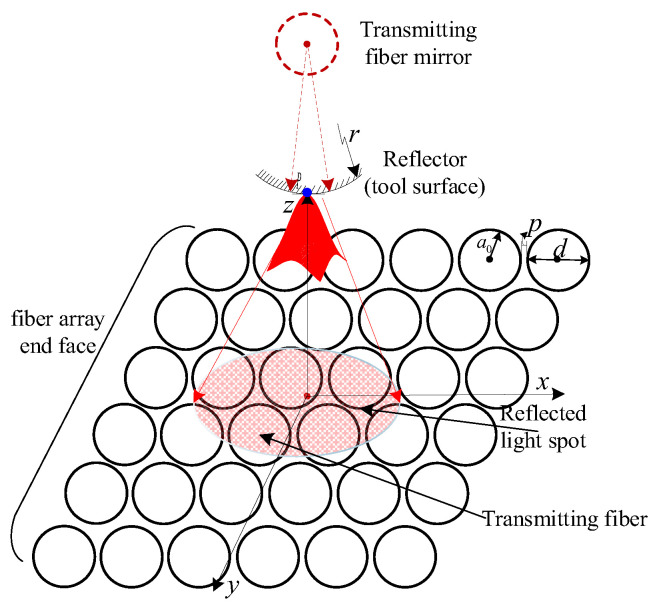
The relative position of tool reflection surface and fiber matrix probe face.

Considering that the motion accuracy of the experimental platform owned by the author is 1 micron, it is assumed that the motion space of the tooltip has a side length of 2 mm. Assuming that the space is labeled with a spacing of 0.1 microns, 8 × 10^12^ points are needed. Therefore, in order to further elaborate the above principles, a 6 × 6 optical fiber array is taken as an example for design and analysis. For the optical array composed of 36 fibers, one fiber is used as the transmitting fiber, and thirty-five are used as the receiving fiber. If the sub-fiber receives reflected light, it is denoted as 1, and if it does not receive reflected light, it is denoted as 0. The intensity of light received by the fiber is converted into a gray value ranging from 0 to 255. Therefore, theoretically, the fiber matrix can realize the marking of 2^35^ × 255 points in the space, and the marking accuracy of an 8 mm^3^ space (8 × 10^12^ points with a spacing of 0.1 microns) can reach 0.1 µm (2^35^ × 255/8 × 10^12^ ≈ 1.09). Theoretically, the more optical fiber arrays, the denser the area is with space markers and the higher the measurement accuracy. The range of tool motion in machining is much smaller than this. Figure 2 shows the relative position relationship between the reflective surface of the tool and the probe end face of the fiber matrix. The radius of the fiber is denoted as a_0_, the spacing of the fiber is p, the numerical aperture of the fiber is NA, and the radius of the measured tooltip is r. It can be seen from Figure 2 that after the tool size and fiber array size are determined, the value of the coding matrix is mainly affected by tool motion.

**Figure 3:**
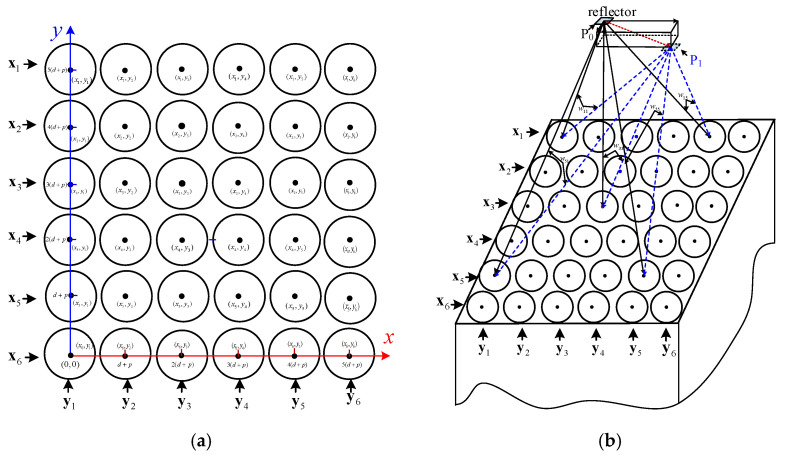
Fiber position matrix and its relation to light intensity matrix. (**a**) Coordinates of the sub-fibers. (**b**) Influence of tool motion on fiber array receiving intensity.

Mark the space coordinates of a reflection point p on the tool as $\left(X_{i},Y_{j},Z_{k}\right)$, where $X_{i}\in \left[0,5\left(d+p\right)\right]$,$\;Y_{j}\in \left[0,5\left(d+p\right)\right]$, $Z_{k}\in \left[z_{1},z_{2}\right]$. Its spatial position can be encoded by both the position matrix and intensity matrix. The position matrix is denoted as M_ab_, as shown in Figure 3a and Equation (1).

(1) $$M_{ab}=\left[\begin{array}{cc} \begin{array}{ccc} \left(x_{1},y_{1}\right) & \left(x_{1},y_{2}\right) & \left(x_{1},y_{3}\right)\\ \left(x_{2},y_{1}\right) & \left(x_{2},y_{2}\right) & \left(x_{2},y_{3}\right)\\ \left(x_{3},y_{1}\right) & \left(x_{3},y_{2}\right) & \left(x_{3},y_{3}\right) \end{array} & \begin{array}{ccc} \left(x_{1},y_{4}\right) & \left(x_{1},y_{5}\right) & \left(x_{1},y_{6}\right)\\ \left(x_{2},y_{4}\right) & \left(x_{2},y_{5}\right) & \left(x_{2},y_{6}\right)\\ \left(x_{3},y_{4}\right) & \left(x_{3},y_{5}\right) & \left(x_{3},y_{6}\right) \end{array}\\ \begin{array}{ccc} \left(x_{4},y_{1}\right) & \left(x_{4},y_{2}\right) & \left(x_{4},y_{3}\right)\\ \left(x_{5},y_{1}\right) & \left(x_{5},y_{2}\right) & \left(x_{5},y_{3}\right)\\ \left(x_{6},y_{1}\right) & \left(x_{6},y_{2}\right) & \left(x_{6},y_{3}\right) \end{array} & \begin{array}{ccc} \left(x_{4},y_{4}\right) & \left(x_{4},y_{5}\right) & \left(x_{4},y_{6}\right)\\ \left(x_{5},y_{4}\right) & \left(x_{5},y_{5}\right) & \left(x_{5},y_{6}\right)\\ \left(x_{6},y_{4}\right) & \left(x_{6},y_{5}\right) & \left(x_{6},y_{6}\right) \end{array} \end{array}\right](a=1,2,\cdots 6,b=1,2,\cdots 6)$$

The light intensity matrix is denoted as ***I****_ab_*, and the intensity of each element depends on the intensity of the light reflected back by each fiber, shown in Equation (2).

(2) $$I_{ab}=\left[\begin{array}{cc} \begin{array}{ccc} I_{\left(x_{1},y_{1}\right)} & I_{\left(x_{1},y_{2}\right)} & I_{\left(x_{1},y_{3}\right)}\\ I_{\left(x_{2},y_{1}\right)} & I_{\left(x_{2},y_{2}\right)} & I_{\left(x_{2},y_{3}\right)}\\ I_{\left(x_{3},y_{1}\right)} & I_{\left(x_{3},y_{2}\right)} & I_{\left(x_{3},y_{3}\right)} \end{array} & \begin{array}{ccc} I_{\left(x_{1},y_{4}\right)} & I_{\left(x_{1},y_{5}\right)} & I_{\left(x_{1},y_{6}\right)}\\ I_{\left(x_{2},y_{4}\right)} & I_{\left(x_{2},y_{5}\right)} & I_{\left(x_{2},y_{6}\right)}\\ I_{\left(x_{3},y_{4}\right)} & I_{\left(x_{3},y_{5}\right)} & I_{\left(x_{3},y_{6}\right)} \end{array}\\ \begin{array}{ccc} I_{\left(x_{4},y_{1}\right)} & I_{\left(x_{4},y_{2}\right)} & I_{\left(x_{4},y_{3}\right)}\\ I_{\left(x_{5},y_{1}\right)} & I_{\left(x_{5},y_{2}\right)} & I_{\left(x_{5},y_{3}\right)}\\ I_{\left(x_{6},y_{1}\right)} & I_{\left(x_{6},y_{2}\right)} & I_{\left(x_{6},y_{3}\right)} \end{array} & \begin{array}{ccc} I_{\left(x_{4},y_{4}\right)} & I_{\left(x_{4},y_{5}\right)} & I_{\left(x_{4},y_{6}\right)}\\ I_{\left(x_{5},y_{4}\right)} & I_{\left(x_{5},y_{5}\right)} & I_{\left(x_{5},y_{6}\right)}\\ I_{\left(x_{6},y_{4}\right)} & I_{\left(x_{6},y_{5}\right)} & I_{\left(x_{6},y_{6}\right)} \end{array} \end{array}\right](a=1,2,\cdots 6,b=1,2,\cdots 6)$$

The distance between the reflected light field and the center of each fiber end face is

$$\omega_{ijk}={\left[(x_{i}-x_{ab}{)}^{2}+(y_{k}-y_{ba}{)}^{2}+z_{k}^{2}\right]}^{1/2}(a=1,2,\cdots 6,b=1,2,\cdots 6)$$

where

$$X_{ab}=\left[\begin{array}{cc} \begin{array}{c} x_{11}\\ x_{21}\\ x_{31}\\ \begin{array}{c} x_{41}\\ \begin{array}{c} x_{51}\\ x_{61} \end{array} \end{array} \end{array} & \begin{array}{cccc} x_{12} & x_{13} & x_{14} & \begin{array}{cc} x_{15} & x_{16} \end{array}\\ x_{22} & x_{23} & x_{24} & \begin{array}{cc} x_{25} & x_{26} \end{array}\\ x_{12} & x_{33} & x_{34} & \begin{array}{cc} x_{35} & x_{36} \end{array}\\ \begin{array}{c} x_{42}\\ \begin{array}{c} x_{52}\\ x_{62} \end{array} \end{array} & \begin{array}{c} x_{43}\\ \begin{array}{c} x_{53}\\ x_{63} \end{array} \end{array} & \begin{array}{c} x_{44}\\ \begin{array}{c} x_{54}\\ x_{64} \end{array} \end{array} & \begin{array}{c} \begin{array}{cc} x_{45} & x_{46} \end{array}\\ \begin{array}{cc} \begin{array}{c} x_{55}\\ x_{65} \end{array} & \begin{array}{c} x_{56}\\ x_{66} \end{array} \end{array} \end{array} \end{array} \end{array}\right](a=1,2,\cdots 6,b=1,2,\cdots 6)$$

$$Y_{ab}=\left[\begin{array}{cc} \begin{array}{c} y_{11}\\ y_{21}\\ y_{31}\\ \begin{array}{c} y_{41}\\ \begin{array}{c} y_{51}\\ y_{61} \end{array} \end{array} \end{array} & \begin{array}{cccc} y_{12} & y_{13} & y_{14} & \begin{array}{cc} y_{15} & y_{16} \end{array}\\ y_{22} & y_{23} & y_{24} & \begin{array}{cc} y_{25} & y_{26} \end{array}\\ y_{12} & y_{33} & y_{34} & \begin{array}{cc} y_{35} & y_{36} \end{array}\\ \begin{array}{c} y_{42}\\ \begin{array}{c} y_{52}\\ y_{62} \end{array} \end{array} & \begin{array}{c} y_{43}\\ \begin{array}{c} y_{53}\\ y_{63} \end{array} \end{array} & \begin{array}{c} y_{44}\\ \begin{array}{c} y_{54}\\ y_{64} \end{array} \end{array} & \begin{array}{c} \begin{array}{cc} y_{45} & y_{46} \end{array}\\ \begin{array}{cc} \begin{array}{c} y_{55}\\ y_{65} \end{array} & \begin{array}{c} y_{56}\\ y_{66} \end{array} \end{array} \end{array} \end{array} \end{array}\right](a=1,2,\cdots 6,b=1,2,\cdots 6)$$

Then, the equivalent radius matrix from the reflected light field to the center of each fiber end face can be obtained:

(3) $${\bar{\omega }}_{ijk}=\sigma a_{0}[1+\zeta (\frac{\omega_{ijk}}{a_{0}}{)}^{3/2}\tan\theta ]$$

(4) $$I_{\left(x_{a},y_{b}\right)}=\frac{K_{0}I_{0}(x_{a},y_{b})}{\pi {\bar{\omega }}_{ijk}}e^{-\frac{{x_{a}}^{2}+{y_{b}}^{2}}{({\bar{\omega }}_{ijk}/2{)}^{2}}}$$

The position matrix ***M*** (optical fiber array position) and the intensity matrix ***I*** (reflected light intensity) together constitute the three-dimensional measurement matrix ***T***, which is as follows:

(5) $$Tp=\hat{p}$$

(6) $$\eta ={\left\|Tp-\hat{p}\right\|}_{2}^{2}$$

where p represents the coordinates of the points based on prior knowledge and $\hat{p}$ is the estimation of the coordinate position of the tool after motion. The solution of Equation (6) is the decoding process of tool space coding, and the specific solution method can be found in the literature [40].

## 3. Experimental Setup

**Figure 4:**
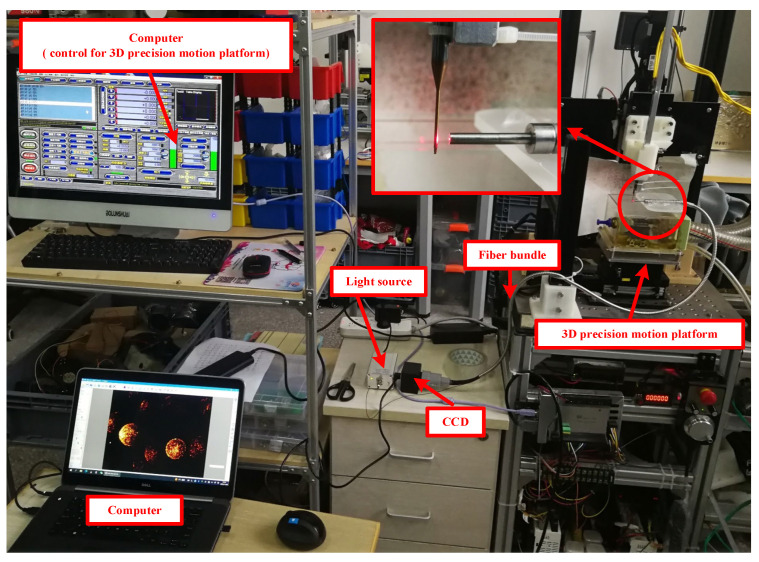
Measurement system setup in 3D precision motion platform.

Figure 4 shows the experimental setup for the verification measurement of the fiber system in x-, y-, and z-directions. The measurement system is mounted on a three-dimensional (3D) precision motion platform. A tungsten steel–coated carbide micro-milling tool (two blades, 4 mm in diameter at the tool shank, and a 16 mm tool overhang length with a 1 mm diameter) is fixed to the 3D precision motion platform. The 3D precision motion platform was developed by the authors, and the motion of the platform in x-, y-, and z-directions are driven by three AC servo motors, respectively (model MM101A2LN08, motion accuracy of 1 micron; A Nidec Group Company, Sankyo, Nagano, Japan).

The optical fiber measurement system mainly consists of four parts: the light source, the fiber array bundle, CCD, and a computer. The light source is a 650 nm red light source with a power of 10 mW. The fiber bundle is an optical array composed of 36 multi-mode fibers (a single fiber diameter of 100 µm, glass material, and a numerical aperture of 0.22). The optical array is square, the section facing the tool is tightly arranged with a side length of 600 microns, the other end with a side length of 0.9 mm connects to the photoelectric converter, and the optical fibers are evenly distributed. The CCD is used for the photoelectric conversion module (SAGA-U500, CCD pixel size of 3.2 µm × 3.2 µm, 5 million pixels, 480 Mb/s).

## 4. Measurement Procedure and Results

### 4.1. Validation of the Optical Fiber System

**Figure 5:**
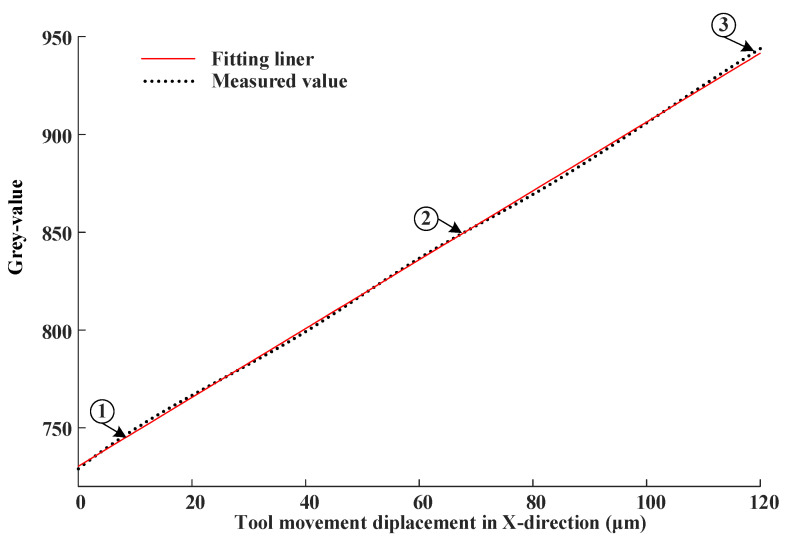
Output in terms of gray values vs. tool displacement change in x-direction.

In order to verify the measurement system, the motion of the tool in x-, y-, and z-directions were measured, respectively. For this, the AC servo motor was driven in steps of 1 µm in a range of 0–120 in the x-direction. In addition, the output in terms of gray values vs. tool displacement change in the x-direction is shown in Figure 5. The process was carried out 15 times. The linear fitting was carried out after taking the mean value of the 15 measurements. Using linear equation fitting, the fitted equation is

(7) $$f\left(x\right)=1.76x+730.5$$

**Figure 6:**
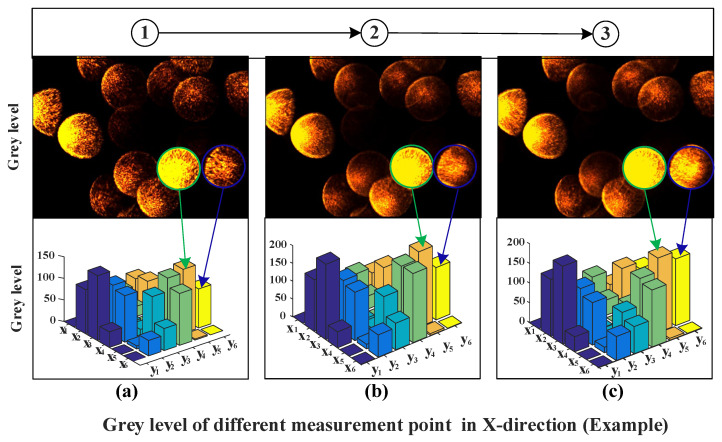
Gray values change examples with the tool displacement in x-direction. (**a**) grey level of point 1 in Figure 5, (**b**) grey level of point 2 in Figure 5, (**c**) grey level of point 3 in Figure 5.

The maximum linear error is 1.5% between the fitting line and measurement data. The maximum linear fitting error of the obtained 15 experimental results was evaluated based on the evaluation method shown in ref. [41]. Additionally, the measurement uncertainty is 0.7%, which means that the measurement uncertainty is 0.84 µm for the range of 120 microns. To further illustrate the variation of fiber array data at different points in the measurement process, three measurement points in the x-direction were taken as examples. The photos captured by the CCD in point 1, point 2, and point 3 in Figure 5 are shown in Figure 6. The gray levels of the fiber array below the respective images are shown in Figure 6 as well. Comparing the optical fiber array image, the difference between the images is very apparent when the tool is located at different positions. The optical fiber images marked by green circles and blue circles in Figure 6 are taken as an example for comparison and analysis. It can be seen that not only does the gray level of the marked fibers of Figure 6a–c change with the tool location, but also their relative sizes change at the same time. Considering the results shown in Figure 5 and Figure 6, the coding of the fiber optic array has significant differences in tool positions in the x-direction.

**Figure 7:**
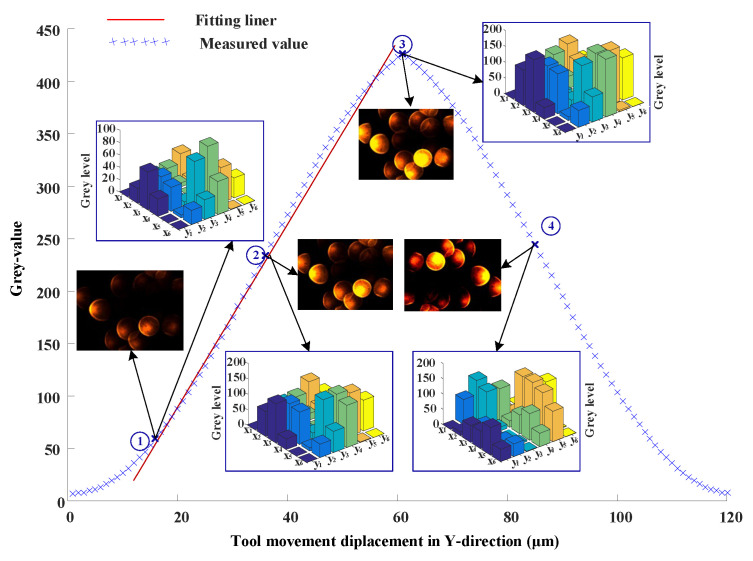
Output in terms of grey values vs. tool displacement change in y-direction.

The AC servo motor in the y-direction was driven in steps of 1 µm in a range of 0–120 µm. Figure 7 shows the output in terms of gray values vs. tool displacement change in the y-direction. Different from Figure 5’s line, there are two parts of the curve in Figure 7. This is because the movements of the tool in the y-direction are parallel to the radial fiber probe. The process was carried out 15 times. The linear fitting was carried out after taking the mean value of 15 measurements. Using linear equation fitting, the fitted equation of the left part in Figure 7 is

(8) $$f_{1}\left(y\right)=9.24y-99.45$$

The maximum linear error is 2.58% between the fitting line and measurement data in the range marked by the fitting line in Figure 7. Similar to the above evaluation method shown in ref. [37], the maximum measurement uncertainty was calculated as 1.24 µm for the range of 120 microns. It can be seen that the signal is strongest when the tool is facing the axis of the fiber bundle. When the tool is far away from the center of the optical field, the optical intensity received by the optical array decreases because of the influence of the numerical aperture of the fiber and the arc of the tool surface. However, although the curves shown on the left and right sides are basically symmetrical in Figure 7, it can be found that there is an obvious difference between measurement points 2 and 4 by comparing the pictures and their gray coding of the two points. In other words, this code not only reflects the tool displacement information but also reflects the tool motion direction information.

**Figure 8:**
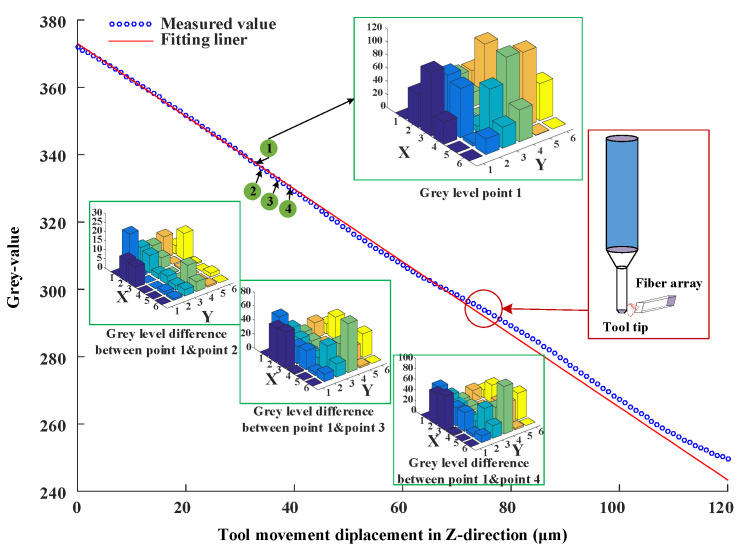
Output in terms of grey values vs. tool displacement change in z-direction.

Similar to the above experiments, the AC servo motor was driven in steps of 1 µm in the z-direction in a range of 0–120 µm. Figure 8 shows the output in terms of gray values vs. tool displacement change in the z-direction. Using linear equation fitting, the fitted equation is

(9) $$f\left(z\right)=-1.05z+371.6$$

The maximum linear error is 2.43% between the whole fitting line and measurement data. However, the maximum linear error is 1.56% on the upper part of the curve shown in Figure 8. There are two reasons for this phenomenon: One is that the area of the tooltip becomes smaller; the other is that the tooltip gradually leaves the space covered by the optical field of the fiber bundle. Similar to the above evaluation method, the maximum measurement uncertainty in the z-direction is calculated as 0.53 µm in the range of 0–65 microns and 1.23 µm in the range of 65–120 microns.

To further observe the change of fiber array coding under the variation of tool motion, the gray coding information of four adjacent measuring points was used for comparative analysis. Figure 8 shows the gray level of measurement point 1, the differences between point 1 and point 2, point 3, and point 4. There are very noticeable differences in the gray levels between point 1 and point 2, point 3, and point 4. In addition, the differences are not only reflected in the gray levels of various measurement points but also reflected in the gray-scale matrix distribution. All these indicate that the tool displacement in the z-direction can be uniquely coded using an optical fiber array.

### 4.2. Validation of the Measurement System for Tool Three-Dimensional Motion in Space

**Figure 9:**
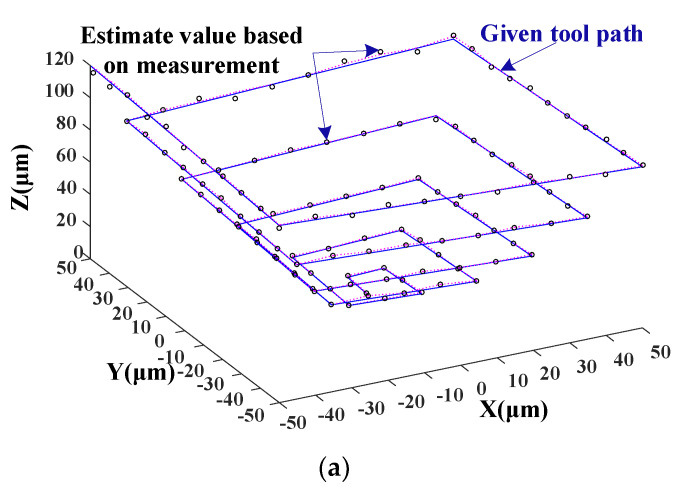

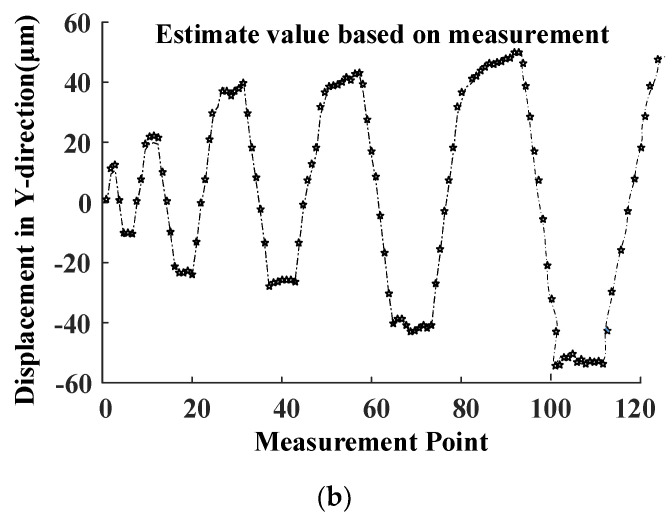
Estimate results of tool’s three-dimensional motion in space. (**a**) Tool motion in space. (**b**) Tool motion in y-direction.

The above experimental results show that the tool displacements in the x-, y-, and z-directions can be uniquely coded using an optical fiber array. To further verify the spatial coding performance of the fiber array, the AC servo motor in the z-direction was driven in steps of 1 µm from 0 to 120 µm. Meanwhile, the AC servo motors in the x- and y-directions were driven in steps of 10 µm moving within a maximum range of 100 µm. Figure 9a shows the tool motion curve driven by three servo motors in space (blue line) and the estimated values (black circles) based on the measurement results of the fiber system. It can be seen that the measured values fit well with the given curve. By comparing the displacement of each measuring point and fixed point from the original shown in Figure 9a, the results show that the maximum error of the measured value deviating from the given value is 3.4%, and based on the evaluation of 15 experimental measurements, the measurement uncertainty is 1.2 µm. Based on the data shown in Figure 9a, as a function of the z-and x-directions, Figure 9b shows the estimated values of tool displacement in the y-direction. According to the tool motion path in Figure 9a, the tool’s motion path in the y-direction within ±50 microns is clearly shown in Figure 9b. Figure 9b shows that the tool moved 10 microns in the positive y-direction firstly (from the first point to the second point), then held there and moved in the other two directions (the second point to the third point). Next, it moved in the negative y-direction in steps of 10 microns (from the third point to the fourth and fifth points). Carefully analyzing the curve shown in Figure 9b, it can be seen that the measured values are in good agreement with the given motion of the tool in the y-direction.

## 5. Conclusions

The vibration signal of the micro-milling tool is weak and complex, which has great potential to harm tool life and workpiece machining quality. Improved vibration measurement methods of traditional milling tools are adopted to detect micro-milling tool vibration signals, such as acceleration sensors, sound pressure sensors, force sensors, laser displacement sensors, and so on. In the application, the existing vibration measurement methods of micro-milling tools do not meet the requirements of online measurement of tool vibration in ultra-precision micro-milling. As most of them are installed far from the tooltip, they are not conducive to the measurements of weak vibrations in micro-milling, and it is difficult to realize the synchronous measurement of the three-dimensional vibration of the tool. In this paper, a multi-fiber coding method is proposed for measuring the three-dimensional displacement of micro-milling tools. The main conclusions include the following:

(1) A 6 × 6 optical fiber array was designed, and a 3D motion platform for micro-milling tools was built to verify the characteristics of the optical fiber measurement system.

(2) The measuring characteristic curves of the measuring system in x-, y-, and z-directions were obtained through experiments. The maximum linear fitting error of the obtained 15 experimental results was evaluated based on the evaluation method. The measurement uncertainty was calculated as 0.84 µm, 1.24 µm, and 0.53 µm in the x-, y-, and z directions, respectively.

(3) The experimental results of tool space motion position measurement show that the maximum measurement error of the measuring system was 3.4%, and the measurement uncertainty was 1.2 µm. This verifies the spatial positioning performance of the measuring system. The experimental results show that the designed system has unique coding characteristics for the tool position in the space of 100 µm^3^.

The current research has limitations: Because of the limitation of the conditions of the authors’ laboratory, the dynamic characteristics of the measurement system could not be verified in detail. This part of the work will be supplemented in a follow-up study. Nevertheless, this paper provides a new possible method for the 3D measurement of micro-milling tools. The method proposed in this paper aims to realize multi-dimensional motion displacement measurement synchronously and avoids the error caused by multi-sensor fusion. Possible applications include the feedback link for active vibration control of the micro-milling tool, micro-milling tool vibration state detection, and so on.

## Data Availability

Data can be made available upon reasonable request.
